# Identification of candidate regions for a novel Usher syndrome type II locus

**Published:** 2008-09-19

**Authors:** Imen Ben Rebeh, Zeineb Benzina, Houria Dhouib, Imen Hadjamor, Mustapha Amyere, Leila Ayadi, Khalil Turki, Bouthaina Hammami, Noureddine Kmiha, Hassen Kammoun, Bochra Hakim, Ilhem Charfedine, Miikka Vikkula, Abdelmonem Ghorbel, Hammadi Ayadi, Saber Masmoudi

**Affiliations:** 1 Unité Cibles pour le Diagnostic et la Thérapie, Centre de Biotechnologie de Sfax, Tunisie; 2 Service d’Ophtalmologie, C.H.U. H. Bourguiba de Sfax, Tunisie; 3 Service d'O.R.L., C.H.U.H. Bourguiba de Sfax, Tunisie; 4 Human Molecular Genetics, de Duve Institute, Université Catholique de Louvain, Brussels, Belgium; 5 Policlinique C.N.S.S. de Sfax, Tunisie; 6 Laboratoire de Génétique Moléculaire Humaine, Faculté de Médecine de Sfax, Tunisie

## Abstract

**Purpose:**

Chronic diseases affecting the inner ear and the retina cause severe impairments to our communication systems. In more than half of the cases, Usher syndrome (USH) is the origin of these double defects. Patients with USH type II (USH2) have retinitis pigmentosa (RP) that develops during puberty, moderate to severe hearing impairment with downsloping pure-tone audiogram, and normal vestibular function. Four loci and three genes are known for USH2. In this study, we proposed to localize the gene responsible for USH2 in a consanguineous family of Tunisian origin.

**Methods:**

Affected members underwent detailed ocular and audiologic characterization. One Tunisian family with USH2 and 45 healthy controls unrelated to the family were recruited. Two affected and six unaffected family members attended our study. DNA samples of eight family members were genotyped with polymorphic markers. Two-point and multipoint LOD scores were calculated using Genehunter software v2.1. Sequencing was used to investigate candidate genes.

**Results:**

Haplotype analysis showed no significant linkage to any known USH gene or locus. A genome-wide screen, using microsatellite markers, was performed, allowing the identification of three homozygous regions in chromosomes 2, 4, and 15. We further confirmed and refined these three regions using microsatellite and single-nucleotide polymorphisms. With recessive mode of inheritance, the highest multipoint LOD score of 1.765 was identified for the candidate regions on chromosomes 4 and 15. The chromosome 15 locus is large (55 Mb), underscoring the limited number of meioses in the consanguineous pedigree. Moreover, the linked, homozygous chromosome 15q alleles, unlike those of the chromosome 2 and 4 loci, are infrequent in the local population. Thus, the data strongly suggest that the novel locus for USH2 is likely to reside on 15q.

**Conclusions:**

Our data provide a basis for the localization and the identification of a novel gene implicated in USH2, most likely localized on 15q.

## Introduction

Usher syndrome (USH) is an autosomal recessive disorders characterized by sensorineural hearing impairment (HI), retinitis pigmentosa (RP), and variable vestibular dysfunction [1]. It is clinically and genetically heterogeneous, and it is categorized into three clinical subtypes. USH type 1 (USH1) is the most severe form. Patients with USH1 suffer from vestibular dysfunction, delayed motor development, congenital sensorineural HI, and RP starting in early childhood. RP is due to photoreceptor degeneration, which occurs from the periphery of the retina to the macula. Night blindness is the first symptom of RP followed by narrowing of the visual field [2]. Those with USH type II (USH2) have moderate to severe congenital sloping HI, normal vestibular function and a late onset of RP. USH type III (USH3) is characterized by variable RP and vestibular dysfunction combined with progressive HI. There are 11 known loci (USH1B-USH1G, USH2A-USH2D, and USH3), and for nine of them, the corresponding genes have been identified: USH1B/*MYO7A*, USH1C/*USH1C*, USH1D/*CDH23*, USH1F/*PCDH15*, USH1G/*SANS*, USH2A/*USH2A*, USH2C/*VLGR1*, USH2D/*WHRN,* and USH3A/*USH3A* (Usher homepage). Mutations in USH2 genes can also manifest as atypical USH [3], as nonsyndromic recessive HI [4], or as nonsyndromic recessive RP [5].

## Methods

### Family and clinical data

**Figure 1:**
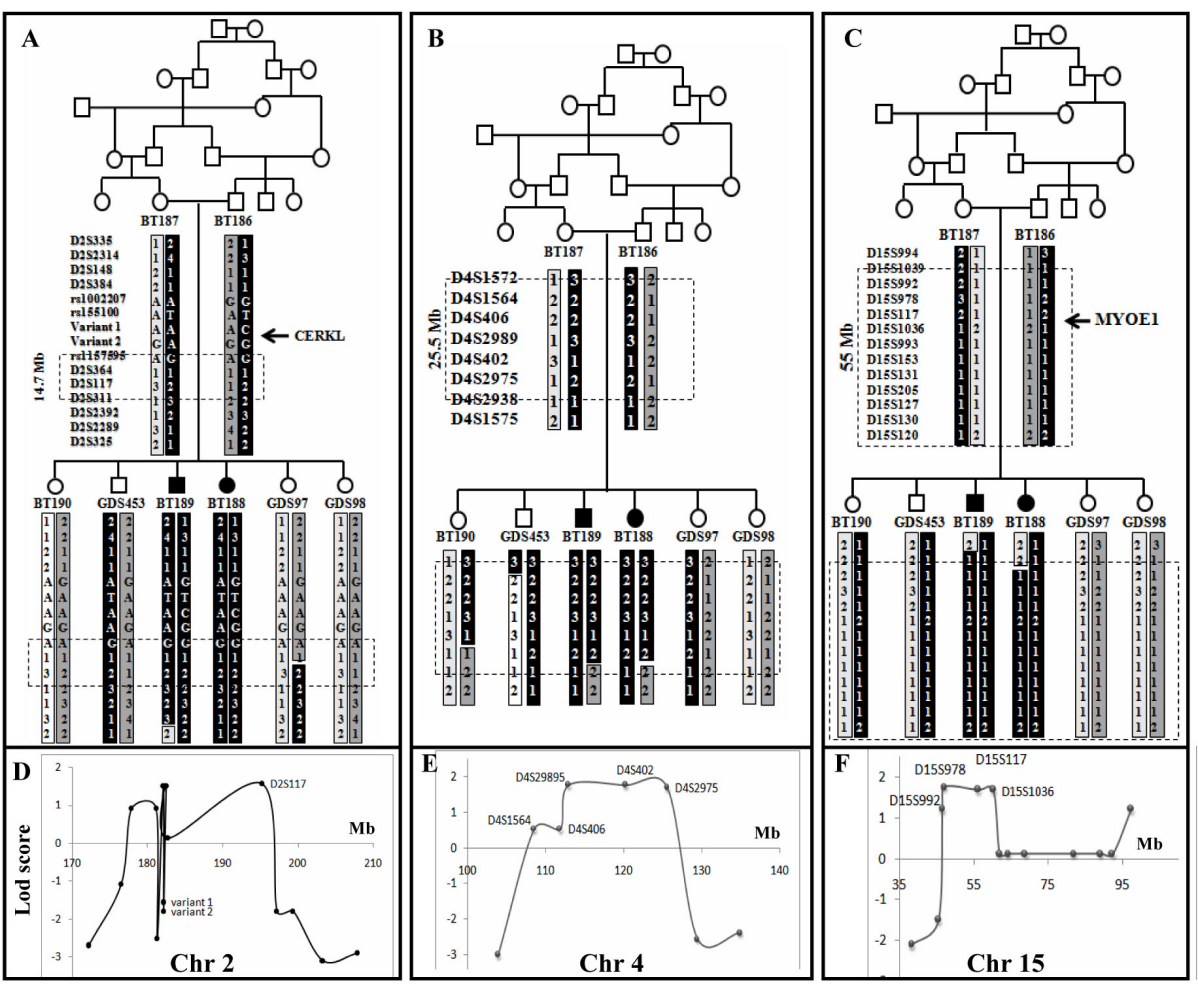
Pedigree, haplotype and statistical data for a Tunisian family segregating Usher type 2 syndrome. **A-C:** In pedigree, the square symbol indicates male, the circle symbol denotes female and black symbols represent affected individuals. Haplotypes for polymorphic markers in three candidate regions on Chromosome 2, 4, and 15 are shown. The disease-linked haplotype is indicated by black bar for markers listed while other haplotypes in gray and white. The critical linkage interval of each candidate region was indicated by box on haplotypes. Analysis of these markers allowed us to refine the boundary of the critical linkage intervals to 14.7 Mb, 25.5 Mb, and 55 Mb respectively. Among interesting candidate genes on chromosome 2 and 15 region two *CERKL* and *MYO1E* were selected for mutation screening. **D-F:** Multipoint lod scores for markers on three candidate regions on Chromosome 2, 4, and 15. Lod scores for the different markers studied were computed using Genehunter software. Maximum lod score of 1.765 was identified for the candidate regions on chromosome 4 between D4S2989 and D4S402 and chromosome 15 between D15S978 and D15S1036. A maximum lod score of 1.51 was found on chromosome 2 between rs155100 and rs1157595. The following abbreviation was used: Mega base (Mb).

In this study, we investigated a Tunisian family with USH2. This family originates from centre of Tunisia. Two affected (1 male and 1 female aged 28 and 18 years, respectively) and six healthy family members (2 males and 4 females aged 21-61 years) attended our study. We also recruited 45 controls (22 males and 23 females aged 26-72 years) from different regions of Tunisia. Written informed consent was obtained from both parents, in accordance with the ethics committee of the University Hospital of Sfax. The pedigree was obtained upon interviews with parents (Figure 1). Clinical history and physical examinations of family members ruled out the implication of environmental factors in the etiology of HI and RP. Eight family members were subjected to audiologic examination, which consisted of otoscopic exploration and pure-tone audiometry. Testing of the vestibular system was performed by electron stagmography. Ocular examinations included fundus ophthalmoscopy, visual field examination, and Ganzfeld-electroretinogram (ERG). Blood samples were collected from eight family members. Genomic DNA was extracted from whole blood following a standard phenol-chloroform method.

### Microsatellite genotyping and homozygosity mapping

For each gene and locus responsible for USH (Usher homepage) at least three microsatellite markers were selected on the basis of their map position (UCSC Genome Browser) and heterozygosity coefficient (HE; minimal HE of 0.7). Fluorescent dye-labeled microsatellite markers were genotyped for all the participating family members. Furthermore, a genome-wide scan was performed using 400 fluorescent dye-labeled microsatellite markers with an average spacing of approximately 10 cM (Prism Linkage Mapping Set, Applied Biosystems, Foster City, CA). We used the True Allele PCR Premix (Applied Biosystems) for PCR reactions according to the manufacturer’s instructions. Fluorescently labeled alleles were analyzed on an ABI Prism 3100-Avant automated DNA sequencer (Applied Biosystems).

We used homozygosity mapping to identify autozygous regions in the two affected children. Two-point and multipoint LOD scores were calculated with Genehunter software V2.1 version, using 100% penetrance, four alleles with equal allele frequencies, and no phenocopies.

### Mutation analysis and single nucleotide polymorphism genotyping

Direct sequencing of candidate genes was performed using primers in the intronic regions (Table 1 and Table 2). Amplified products were directly sequenced using an ABI 3100-Avant automated DNA sequencer and Big Dye Terminator Sequencing V3.1 Kit (Applied Biosystems).

**Table 1:** Primer pairs used to sequence coding exons of *CERKL*.

**Exon**	**Forward sequence**	**Reverse sequence**	**PCR size (bp)**
1	GTGCTGGACTGGGTCAGG	CAAAAGCTCGTGGGTGTAGG	490
2	CCCCAGTGTCTGTTGTTCCT	TCAAGGAAACTGGGCTGATT	356
3	TGTGTCATTTTAAAGGGAAAGAAA	TTCCCAAGTTTGCATTAAGGA	295
4	TTTGCCAGAACAAGTTAAAAAGTG	TGAACAAGATAGAGCCAAAGTAA	273
5	CCCATTGGTTAACTTGTCTGTG	CACATCAGTCCAACACTTTAGCA	295
6	GGTACATGTGAGCAGTTATGCAC	TAGTGGGGATGCCAGAAGTC	399
7	AAAAGCAAATGTTAGTTTGAACACA	AGAGACAAAGAACCTGCCTTTT	249
8,9	GCTCTCTTATGTTTGCTGTTTTGA	TCTGATCAATTGTTTGTCAGAATG	461
10,11	GCGCGCGTTATCTGTTTTAT	CAGTTAATTGGATACCCTGGAAA	352
12	CATGGTGATTTATCTATCTTGTCCA	CAATTCTTGCAGCATCTTTTTC	299
13	CTCAAAGCTATTAAAATGTCAGCA	AACCAACTGCCTGCTTTGAT	400
14	TATTTGGCATTGGCATTGTG	GGTTTAAAGCATGGCCACAT	222

**Table 2:** Primer pairs used to sequence coding exons of *MYO1E*.

**Exon**	**Forward sequence**	**Reverse sequence**	**PCR size (bp)**
1	TACGGTTTCCCTGAGGAGTG	CGCGTCCACCTTCTCCAC	588
2	TCTGCACTGCTCTTTCTGCT	AACTCCTGCCTTAGCCTTCC	395
3	TTGTGAATTCTTGATAACATCTGG	TCAAGAAAAACCATGTCTGCAT	248
4	TAGTGCACGATTCGTTTCCA	CCTGCTTGCTACTCAGACACA	355
5	GTTTTGTGTGATGGGGGAGA	CCAGTGTCTTTTCTGTGGAAGA	271
6	GGCCCCTCACCTTAATGC	TATGTGAAAGGCTCCCATTT	299
7	AGGATGCAGGAGTGACTTCG	GAAAGAGGCGGACATTTCA	320
8	TGTGACTGCACAACCCAATC	TGCCACAGAGGACATGTAGA	440
9	CCCGTGATTGTGCCTTCTAT	CGCACCCAGCCTACTAGTTT	396
10,11	GTCCTCTGTTTCCTGCAAGC	TTGTTTTTGCATTGCCTAGA	292
12	AAGGAGTTCACTGCCATGCT	GCCACAATGGCATATGGTTT	684
13	TGTTCCTTTCCTGTTACCTCTT	TCAGAGTTGTCACTTTGCCTGT	359
14	GCCATGACAGCTTTGGTTTT	AGGAACACACCACCACACC	299
15	CCCTTCACCCCATCCTCTA	CAGGGGTGCAGTTCCTTACT	243
16	TGCTTAACGAGCAAATTGTCA	AAGACATGTGCGGACAACTG	349
17	TCCCTACAGCTTGGAACTGG	GTACGCTTGAAGTGGGTGAA	286
18	TTCGAACGCTGGTAAACAGA	CAACATTGATGGCATGAAGC	398
19,20	TCCCGTGTGTGTCATTGTCT	AACGAACACATTCTGATTTGG	708
21	CCTCCGAAAGTACTGGGATT	TCCTCCTGGCTGTTTGGAAC	304
22	TCATTGTTGTTGGTTTTGTTTG	GCGATCAAGACCCCTTTTTA	366
23	CCCTGCTCCTGGTGTAGATT	GTGCACATGTTTGCAGCATT	374
24	TCCACCTGAGAGCTGGAATC	TCCAGATTTAGTGGTCCCAGA	250
25	TTCAAATGCGGAAATTGAGAC	ATGATGGAGATGGAGCTTGC	383
26	AAGGATGGAGCTGGATTTGA	AGCAATGTGACTGCATGCTC	347

We also analyzed by direct sequencing three single nucleotide polymorphism (SNP) markers (rs155100, rs1157595, and rs1002207; dbSNP) spanning the ceramide kinase-like gene (*CERKL*) to check their cosegregation with the disease before proceeding to mutation screening. Primers and conditions were previously described [6].

## Results

### Family and clinical data

**Figure 2:**
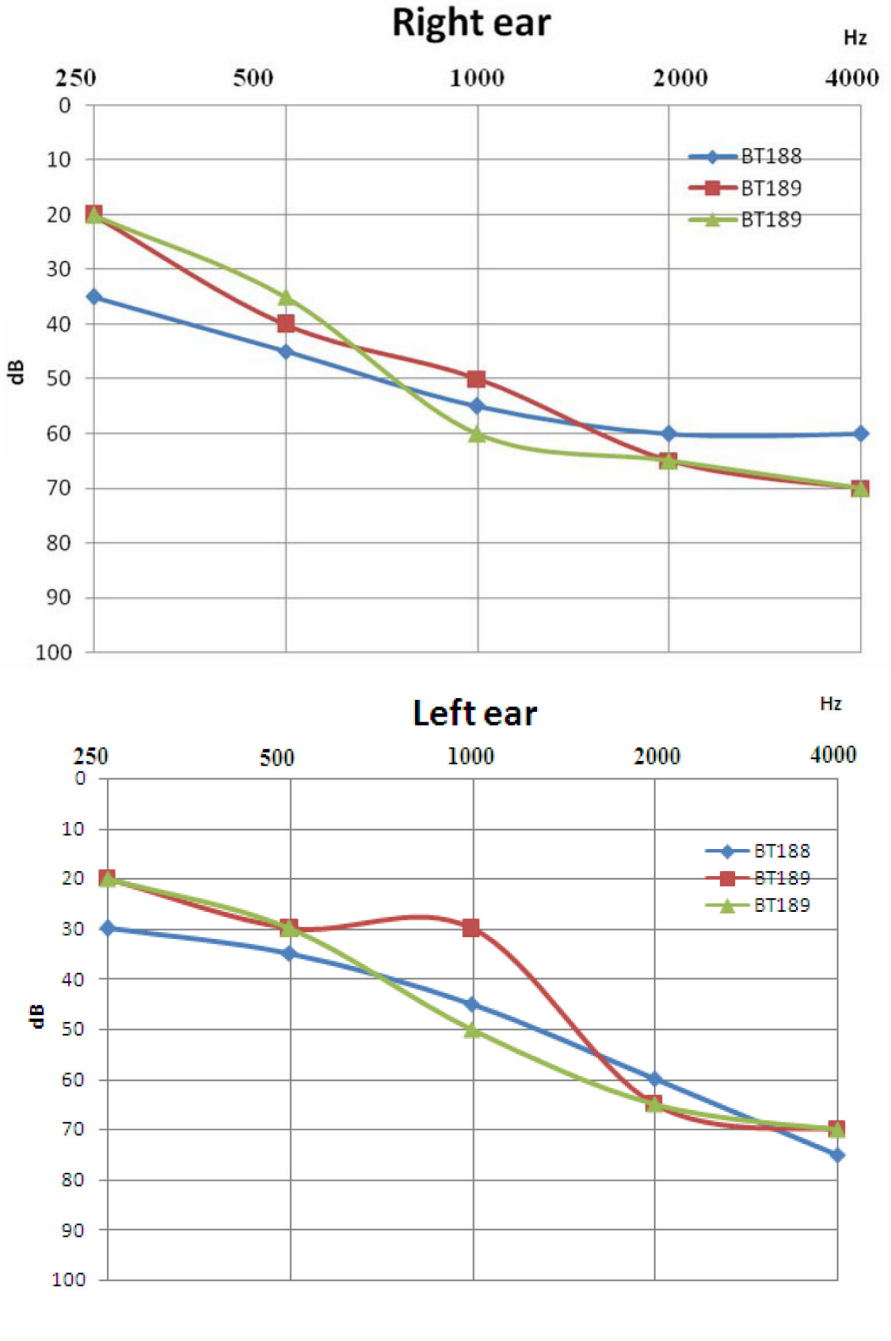
Serial audiograms of two affected members (BT188 and BT189) shown for the right (R) and left (L) ears separately. Pure tone air-conduction threshold (y-axis) is expressed in decibels (dB). The blue one represents the audiogram from 18-year-old BT188. Both the red and the green represent the audiograms for the patient BT189. The red audiogram was made when he was 24 whereas the second was made at the age of 28. BT188 was not available for audiometric test at the beginning of the study. Audiometric test showed a moderate sloping bilateral sensorineural HI in these two usher patients. The green and the red audiograms for the patient BT189 showed that there is no progression of hearing loss at an interval of four years.

**Figure 3:**
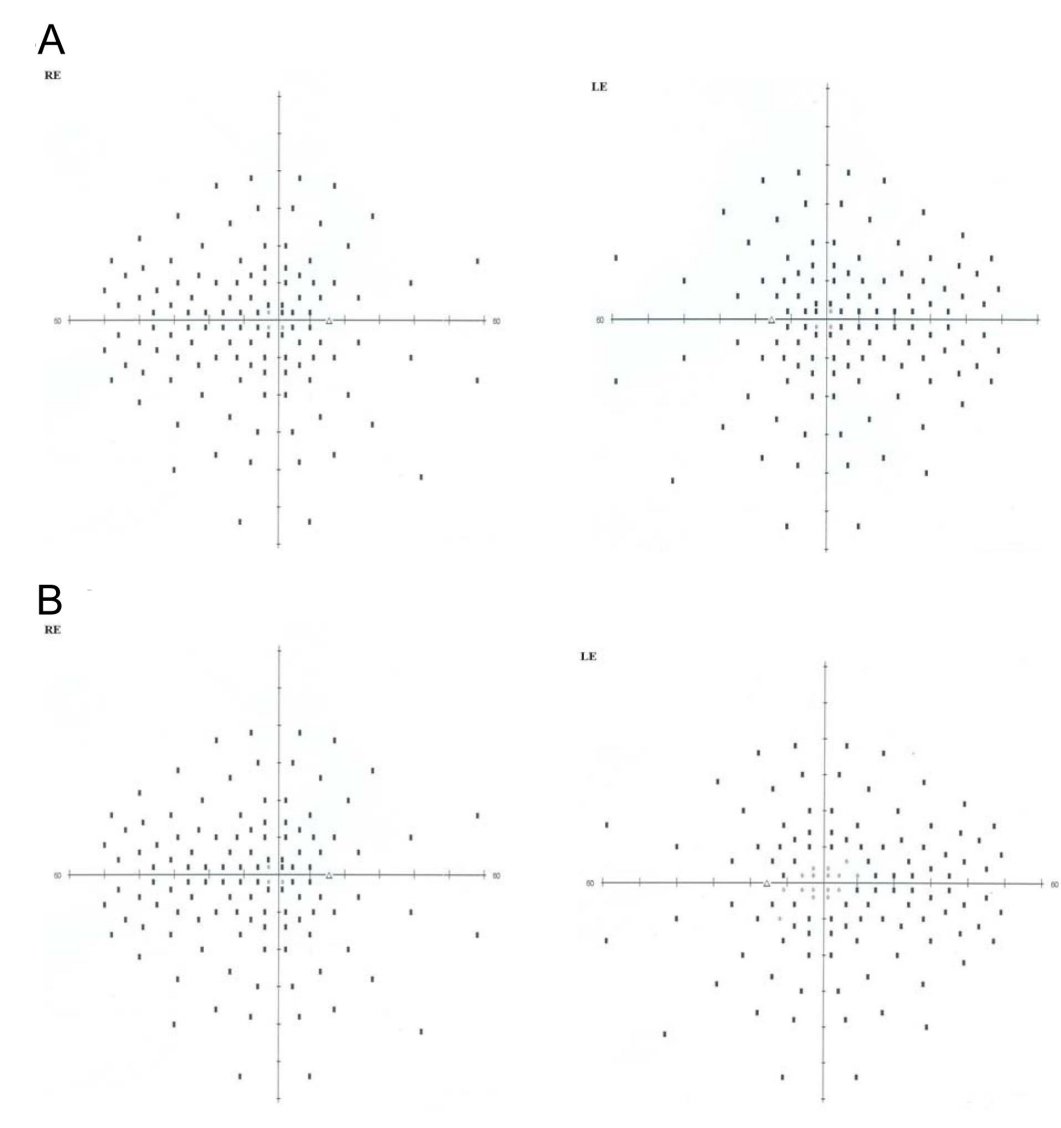
Visual field test results obtained on the right (RE) and the left eye (LE) of the two patients BT188 and BT189. **A:** Result of measuring the visual fields on BT189 at 28 years of age. **B:** Result of measuring the visual fields on BT188 at the age of 18 years. A series of random lights of different intensities are flashed in the peripheral field of vision of both patients. When they perceive the computer-generated light suddenly appearing in their field of view they press a button to indicate their responses, then we see this spot (Dot see). If the patient is unable to see the light in an appropriate portion of his field of view, then we see on the computer a spot (Dot don’t see) indicating vision loss. Visual field loss was more severe in the older brother BT189. But in both patients, the nasal and temporal fields were not preserved, and only the central field was maintained.

**Figure 4:**
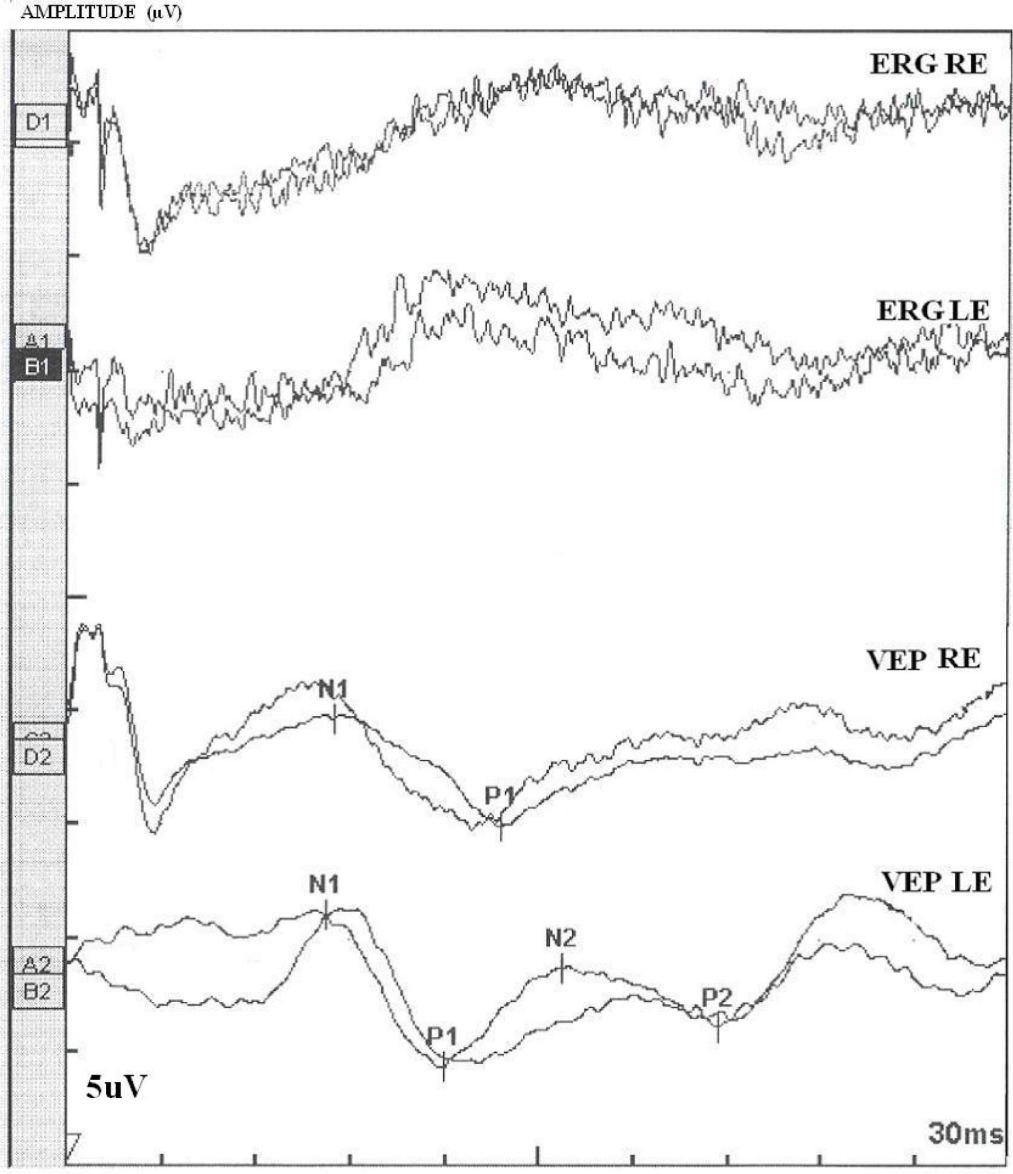
Ganzfeld-Electroretinogram of the right and left eyes of the patient BT189. The following abbreviations were used: Left eye (LE), right eye (RE), electroretinogram (ERG), visual-evoked potentials (VEP), positive peak (P1 and P2), negative peak (N1 and N2). The ERG and the VEP tests the function of the visual pathway from the retina (ERG) to the occipital cortex (VEP). These tests were conducted by placing a standard ERG device attached to the skin on 2 mm above the orbit. VEPs were recorded simultaneously from electrode attached to the occipital scalp 2 mm above the region on the midsaggital plane. An electrode placed on the fore head provided a ground. The results can be directly related to the part of a visual field that might be defective. This is based on the anatomical relationship of the retinal images and the visual field. After dark adaptation for 30 min, the doctor will place anesthetic drops in the patient's eye and place a contact lens on the surface of the eye. Once the contact lens is in place, a series of blue, red and white lights will be shown to the patient. The VEP is an evoked electrophysiological potential that can be extracted, using signal averaging, from the electroencephalographic activity recorded at the scalp. Both ERG and VEP were differentially amplified band pass filtred (0,1,30 Hz), recorded over 300 ms epochs, and signal average. 2 trials were given. The visual evoked potential to flash stimulation consists of a series of negative and positive waves. The earliest detectable response has a peak latency of approximately 30ms post-stimulus. For the flash VEP, the most robust components are the N2 and P2 peaks. Measurements of P2 amplitude should be made from the positive P2 peak at around 207.3 ms. The ERG recorded in BT189 showed an absence of responses. While the VEP showed a normal responses in both eyes. These traces confirm the evidence of a significant bilateral global retinal degeneration. Only cone flicker responses of less than 15% of the normal mean were recordable under photopic conditions while all other responses were below noise level, a typical finding for patients with retinitis pigmentosa.

The pedigree in Figure 1 displays a consanguineous Tunisian family segregating USH based on clinical history and audiometric and ophthalmologic tests. Audiometric test showed a moderate sloping bilateral sensorineural HI in USH patients (Figure 2). Severity of HI was similar in patients BT188 and BT189. Parents reported that HI was first noted in BT188 when the child was six years old, but observed it in BT189, when the older sibling was ten years old. For patient BT189 two audiograms were made at four-year intervals with no change in the profile (Figure 2). The father (60 years) had high frequency HI caused by bilateral presbycusis. No vestibular dysfunction was detected in both patients (BT188 and BT189) using the caloric test, nor was there any history of a delay in the age of walking. Patient BT189 reported having night blindness problem beginning at the age of 13 years. Fundus examination at in BT188 at age 14 and in BT189 at age 24 detected severe retinal degeneration. Visual fields (Goldmann targets III/4e) were significantly reduced to 5° concentric field and temporal island fields in BT189 for both eyes and 5° and 10° respectively in left and right eye in BT188. In BT189 and BT188, the nasal and temporal fields were not preserved, and only central field was maintained (Figure 3). The Ganzfeld-ERG recorded in BT189 showed an almost normal response flash visual-evoked potential in both eyes and a significant bilateral global retinal degeneration. Only cone flicker responses of less than 15% of the normal mean were recordable under photopic conditions while all other responses were below noise level (BT189), a typical finding for patients with RP (Figure 4). Nystagmus was noted in patient BT188 since her first examination at age 14 years. No other abnormalities were observed in these two patients. Taken together, the clinical signs observed in affected subjects indicate a form of USH2.

### Genome-wide screening and homozygosity mapping

To localize the causative gene, we performed linkage analysis using polymorphic microsatellite markers bordering all described USH loci and genes. The USH phenotype segregating in the family was not found to be linked to the published USH loci. Table 3 shows statistical evidence for exclusion of *USH2* genes. Therefore, a genome-wide screen using microsatellite markers was performed. Linkage was found with four markers D2S117 (2q32.3), D4S402 (4q26), D15S978 (15q21.1), and D15S117 (15q22.1). Additional markers were genotyped in all three regions to define the critical intervals. The homozygous region in chromosome 2 was delimited by two informative markers rs1002207 and D2S311; the chromosome 4 region was bordered by D4S1572 and D4S2938; and the chromosome 15 region stretched from D15S1039 to D15S120 (Figure 1). Analysis of these markers allowed us to refine the boundary of the critical linkage intervals respectively to 16 Mb, 25.5 Mb, and 55 Mb. In the CH15 region, six polymorphic microsatellite markers were found to be noninformative in the family (Figure 1). Investigation of the polymorphism of these repeats in the Tunisian population was performed in 40 unrelated individuals from different areas. Our results demonstrated that these microsatellites display a high degree of genetic polymorphism in the general Tunisian population. Microsatellite marker heterozygosity values were estimated using HET software version 1.8 and are as follows: 0.61 for D15S993, 0.89 for D15S153, 0.84 for D15S131, 0.86 for D15S205, 0.85 for D15S127 and 0.83 for D15S130. To rule out chromosome 15 aberrations, we performed G banded karyotype analysis on Phytohemagglutinin (PHA)-stimulated blood culture using standard procedures. Chromosome analysis of patient BT189 showed normal karyotype (data not shown).

**Table 3:** Two point LOD scores calculated for microsatellites bordering all described USH2 gene regions.

**Gene**	**Marker**	**Recombination fraction (q)**				
		**0**	**0.1**	**0.2**	**0.3**	**0.4**
*USH2A*	D1S425	-∞	-0.423	-0.096	-0.01	0.001
	D1S2827	-2.828	0.024	0.071	0.042	0.012
	D1S213	-2.832	0.022	0.07	0.042	0.012
*VLGR1*	D5S428	-∞	-0.165	-0.073	-0.034	-0.009
	D5S618	-∞	-0.165	-0.073	-0.034	-0.009
	D5S644	-∞	-0.251	-0.117	-0.051	-0.013
*WHRN*	D9S1677	0.328	0.206	0.109	0.046	0.013
	D9S1776	-∞	-0.536	-0.193	-0.067	-0.014
	D9S1682	-∞	-0.119	-0.039	-0.018	-0.005

Two-point LOD scores for the different markers studied were computed using Genehunter software. Close linkage to the known USH genes on chromosomes 1, 5, and 9 was excluded with negative 2-point LOD scores.

We genotyped markers located in the three candidate regions in 40 healthy unrelated Tunisian individuals for more accurate estimation of allele frequencies and to determine the best candidate region. In the first region, we analyzed four markers (D2S148, D2S384, D2S364, and D2S117). In the first region, homozygous alleles were predominantly present in the population and the allele frequencies were 0.35 (D2S148), 0.125 (D2S384), 0.311 (D2S364), and 0.203 (D2S117). For the second region, three markers were analyzed and the frequencies of linked alleles were as follows: 0.025 for D4S2989, 0.122 for D4S402, and 0.125 for D4S2975. In contrast, the homozygous alleles of the chromosome 15 region were not frequent in controls. Allele frequencies of the polymorphic markers D15S992, D15S978, D15S117, and D15S1036 were assumed to be 0.05, 0.05, 0.1, and 0.075, respectively. These results suggest that the disease locus is most probably on chromosome 15. Multipoint LOD scores were calculated for the family data using Genehunter software. Maximum LOD scores (1.765 at θ=0) were identified for the candidate regions on chromosome 4 between D4S2989 and D4S2975, and chromosome 15 between D15S978 and D15S1036. On chromosome 2, a maximum LOD score of 1.51 was found for D2S117 microsatellite marker.

### Candidate gene screening

The evaluation of the three homozygous regions revealed a large number of known and hypothetical genes (UCSC Genome Browser). More than 100 candidate genes in these three regions are expressed in the inner ear and in the retina. Although the region on chromosome 2 was not the best candidate locus (the lower LOD score and homozygous alleles of each linked marker are common in Tunisian population), we chose to investigate the *CERKL* gene, encoding a ceramide kinase, as candidate since it has been described to cause nonsyndromic autosomal recessive RP (*RP26*) [7]. The basis of this choice is that mutations in *USH2A* are responsible for USH2 as well as nonsyndromic recessive RP [5]. SNP (rs1157595 and rs155100) genotyping was compatible with linkage of the *CERKL* gene by cosegregation and homozygosity criteria (Figure 1). However, BT188 and BT189 were heterozygous for the rs1002207 (C/T), which was located at 0.8 Mb from *CERKL* gene. We screened this gene for mutations. Two affected children (BT188 and BT189) were compound heterozygous for two novel variants (Figure 1). The first change was a G>A (c.1073+34G>A) transition at position 34 from the donor splicing site of intron 8. The second was a c.242A>C transversion in exon 2, which leads to p.Asp81Ala substitution. Molecular modeling of the N-terminal region showed that the mutation p.Asp81Ala has no structural effect [8-13]. We detected this variant at heterozygous state in 2 out of 45 Tunisian controls. Taken together, these results exclude this variation to cause any functional defect on the encoded enzyme. Therefore, the locus on chromosome 2 was reduced to 14.7 Mb (Figure 1).

We also screened for mutations in another gene, *MYO1E*, encoding an unconventional myosin and representing a very good candidate on chromosome 15 locus. No nucleotide variant was detected in this gene.

## Discussion

In this paper, we report a consanguineous family of Tunisian origin, composed of two affected children with USH. On the whole, the clinical signs observed in affected subjects from this family were indicative of USH2. USH2 is characterized by moderate to severe HI, and onset of RP in the second decade of life. Vestibular function is not impaired in this subtype. Subtle variations within the USH2 phenotype have been observed in several studies. Liu et al. [4] showed that mutations in the USH2A gene were present at homozygous state not only in typical USH2 patients, but also in USH3-like patients who present with late onset progressive deafness that is occasionally associated with vestibular dysfunction. The p.R334W mutation either causes USH2 or atypical USH [14]. Nystagmus was also described in USH2 patients [15].

This consanguineous Tunisian family displayed no evidence of linkage to any known USH locus. A genome-wide genotyping was performed and revealed three homozygous regions on chromosomes 2q31.3–33.1, 4q24–28.2, and 15q21–15qter. The highest LOD scores were identified for the regions on chromosome 4 and 15. The determination of population frequencies of the homozygous alleles of each linked marker in these three regions showed that only the homozygous alleles of chromosome 15 were rarely present in 40 control Tunisian individuals. More controls (45) were used to check for the novel variant on *CERKL* gene. On the basis of these results, we believe chromosome 15 locus is the most likely locus for the defective gene. This region colocalizes with an autosomal recessive nonsyndromic HI locus (*DFNB48*) mapped to 15q23-q25.1 in five large Pakistani families [16]. Among interesting candidate genes on chromosome 15 region, one gene, *MYO1E*, was selected. Myosins are motor proteins that hydrolyze ATP and translocate along actin filaments [17]. Indeed, the involvement of unconventional myosins in hereditary HI is well documented [18]. Mutations of myosins IA, IIIA, VI, VIIA, and XVA are associated with HI in humans [19-23]. Mutations in *MYO7A* have been reported essentially in families with USH1 but also can lead to atypical USH [24]. *MYO1E* is a member of a *Myosin I* isozyme which are essential for hair cells, the sensory cell of inner ear. All eight Myosin I isozymes are expressed in rodent auditory and vestibular epithelia. Three Myosin I isosymes *Myo1b*, *Myo1c*, and *Myo1e*, are expressed at birth in cochlea and vestibular organs. In mouse, *Myo1e* is expressed in hair cell of the auditory and vestibular epithelia. [25]. This isozyme was enriched in the cuticular plate. Myosin Ie may mediate adaptation of mechanoelectrical transduction. All exons and the flanking sequences of the *MYO1E* gene were sequenced in patients and were found to be negative for functional sequence variants.

As the chromosome 15 interval is large and no more information can be obtained from this family to reduce the size of this locus, other families with USH2, even if small, would be useful to identify the novel gene.
